# Integrating Wearables Into a Hybrid Care Model: Continuous Remote Monitoring of a Diabetic Patient With Frequent Premature Ventricular Contractions Using an ŌURA Ring

**DOI:** 10.7759/cureus.86174

**Published:** 2025-06-16

**Authors:** Hala Zakaria, Idalys Roman, Ihsan Almarzooqi, Ali Hashemi

**Affiliations:** 1 Research, GluCare.Health, Dubai, ARE; 2 Cardiology, GluCare.Health, Dubai, ARE

**Keywords:** arrhythmias, cardiovascular medicine, digital wearable, hybrid model of care, remote monitoring

## Abstract

This case report discusses the management of a male in his early 40s with type 2 diabetes and a history of smoking, evaluated for cardiac risk prior to beginning a half-marathon training program. The patient wore an ŌURA ring before, during, and after a treadmill stress test; however, data review was conducted retrospectively. Although asymptomatic, the patient exhibited frequent premature ventricular contractions and abnormal heart rate recovery during the test, prompting further evaluation. Managed through a hybrid care model that integrated traditional clinical assessment with digital health monitoring, this case highlights the valuable role of wearable technology in identifying and managing cardiac arrhythmias. It also demonstrates how real-time and remote data from the ŌURA ring enhanced clinical decision-making and patient care.

## Introduction

Premature ventricular contractions (PVCs) are extrasystolic heartbeats originating from the ventricular myocardium and represent one of the most common types of arrhythmias observed in both healthy individuals and those with underlying heart disease [1]. While often benign, their presence can also signal more serious cardiac pathology, particularly in patients with established risk factors such as type 2 diabetes mellitus (T2DM) and tobacco use [1]. The diagnostic yield for detecting PVCs is strongly influenced by the duration of monitoring; in asymptomatic individuals, short-term Holter monitoring (24-48 hours) has a low diagnostic yield of approximately 1-3% for identifying intermittent PVCs [2].

T2DM is associated with a heightened risk of cardiovascular disease, including arrhythmias and heart failure [3]. The underlying pathophysiological mechanisms linking T2DM to arrhythmias include autonomic dysfunction, electrolyte disturbances, and structural and functional cardiac remodeling [3]. In addition, smoking further increases cardiovascular risk by promoting oxidative stress and systemic inflammation, which can worsen myocardial injury and heighten arrhythmic susceptibility [4].

The adoption of digital wearable technologies for cardiac monitoring has increased significantly, especially among individuals with multiple risk factors. Devices such as the ŌURA ring, which tracks physiological parameters including heart rate variability (HRV), provide real-time insights into cardiac status and can support clinicians within hybrid care models [5]. These models combine continuous remote monitoring with in-person clinical care, enhancing patient outcomes and allowing for timely treatment adjustments [6,7]. In this context, “real-time” refers to daily synced summaries rather than continuous ECG-grade data. Still, such continuous monitoring can identify subtle deviations from a patient’s baseline - changes that might go undetected during periodic assessments like ECGs or stress tests - thus enabling earlier detection and intervention strategies [8].

The integration of wearable technology represents a major advancement in continuous health monitoring, particularly for high-risk patients who benefit from real-time cardiac assessments. This case underscores the importance of incorporating digital biomarkers from wearable devices into routine clinical practice. By strengthening hybrid care models, such integration offers a more comprehensive view of a patient’s health that complements and enhances traditional, in-clinic evaluations. Moreover, this case highlights the potential role of devices like the ŌURA ring in standard cardiac risk assessments and their promise in improving clinical outcomes in high-risk populations.

## Case presentation

Patient information

A male in his early 40s with T2DM and a history of smoking visited the GluCare.Health clinic for a cardiac risk assessment before resuming training for a half marathon. He was already being monitored under GluCare’s hybrid care model and had been issued an ŌURA ring as part of his diabetes management plan. The new digital biomarkers transmitted from the ŌURA ring were remotely monitored by the healthcare team.

Although the patient’s usual resting heart rate during prior digital monitoring averaged around 90 bpm and no abnormalities were detected on his initial in-clinic ECG, a treadmill stress test was performed due to his risk factors. During the test, he appeared anxious, and his ECG showed isolated PVCs and an elevated heart rate of 103 bpm. The stress test revealed frequent PVCs, particularly in the early stages, which diminished at peak exercise but reappeared rapidly during the recovery phase. His heart rate recovery was abnormal, decreasing by less than 12 bpm in the first minute post-exercise and remaining elevated at around 120-125 bpm with a bigeminy rhythm for over 10 minutes.

This prompted further evaluation using retrospective data from his ŌURA ring over the previous two weeks. The review showed a persistently elevated daytime heart rate and a downward trend in HRV compared to his baseline during the week leading up to the stress test. The patient had been wearing the ŌURA ring continuously before, during, and after the treadmill stress test as part of his ongoing diabetes care. Although the device was not used in real-time during the test, the clinical team retrospectively reviewed the wearable data after identifying frequent PVCs on the ECG. This review revealed changes that had not been captured during standard in-clinic assessments, such as ECG and vitals, suggesting an underlying autonomic imbalance and potential arrhythmic burden.

**Figure 1:**
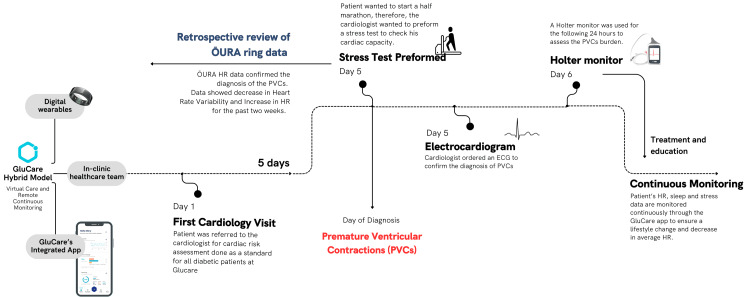
Flow of events in the case presentation using GluCare’s hybrid care model and the ŌURA ring

Given that increased PVC frequency, particularly a burden greater than 10%, has been associated with adverse cardiac remodeling and heart failure risk, the clinical team proceeded with 24-hour Holter monitoring to assess PVC burden and complexity. These findings raised concern because frequent PVCs may contribute to biventricular dysfunction and dilation. The ŌURA ring data revealed potential issues that had been missed during previous in-clinic testing, as the patient had been asymptomatic and showed a normal ECG the day before the stress test. Figure 1 illustrates the flow of events in the case.

Clinical findings

Treadmill Stress Test

The patient underwent a treadmill exercise test using the standard BRUCE protocol, reaching a maximum of stage 4 and completing 10 minutes and 10 seconds of exercise, which corresponded to a workload of 11.8 METs. At rest, his heart rate was 102 bpm, which increased to a peak of 176 bpm during exercise, 99% of the age-predicted maximal heart rate. This elevated heart rate was attributed to pretest anxiety and did not reflect his typical baseline, which had been consistently lower based on wearable monitoring. The baseline ECG revealed PVCs, alternating between isolated, bigeminy, and trigeminy rhythms. These PVCs persisted throughout the first three stages of the exercise test but were not present at peak exertion. During the recovery phase, frequent PVCs reemerged, predominantly in a bigeminy pattern; however, the patient remained asymptomatic.

His blood pressure, initially 120/80 mmHg at rest, rose to a maximum of 160/80 mmHg during exertion. The test was terminated due to fatigue and leg pain, with no chest pain or angina symptoms reported. An ascending, nonspecific upsloping ST depression was observed at peak exercise. This finding is a common physiological response in younger individuals and is not typically considered indicative of myocardial ischemia in the absence of chest pain or other ECG abnormalities. The overall test results were negative for stress-induced ischemia but demonstrated abnormal heart rate recovery and frequent PVCs, particularly during the recovery phase.

ŌURA Ring Data Analysis

**Figure 2:**
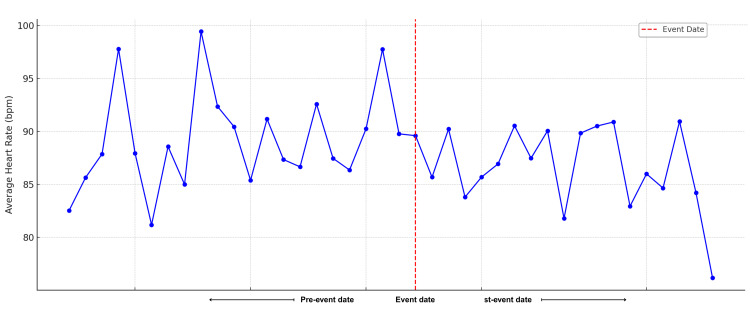
Trend in average daily heart rate two weeks before and after diagnosis

The dataset analyzed included daily average heart rates from June 27 to August 9, 2024, spanning three weeks before and after the stress test conducted on July 18, 2024 - the date the cardiologist established the diagnosis (Figure 2). In the weeks preceding this date, the ŌURA ring data revealed notable variability in daily average heart rates, suggesting underlying physiological instability. This trend likely reflected the emergence of the patient’s condition, as shifts in stress or activity levels were captured through wearable monitoring. While the ŌURA data did not provide a diagnosis, it raised clinical suspicion and prompted further evaluation through ECG and Holter monitoring, which confirmed the presence and burden of PVCs. This underscores the role of wearables as adjunctive tools within the clinical decision-making hierarchy, rather than as standalone diagnostic devices.

A persistently elevated heart rate above 110 bpm at rest for more than 10 minutes, without exertion, has been proposed as a threshold for further investigation, especially in high-risk populations [8,9]. In this case, a progressive decline in HRV, specifically a drop in RMSSD below 20 ms and reduced total variability over a seven-day period, was interpreted as a marker of physiological instability. These changes were consistent with heightened sympathetic activity or autonomic imbalance. ECG testing, performed by the cardiologist, provided definitive confirmation by recording the heart’s electrical activity and verifying the PVCs that had been initially suspected based on the wearable data. While the ŌURA ring does not detect arrhythmias like PVCs, it supplies surrogate physiological markers, such as heart rate and HRV, that can support early clinical insight and guide timely diagnostic escalation.

ECG

**Figure 3:**
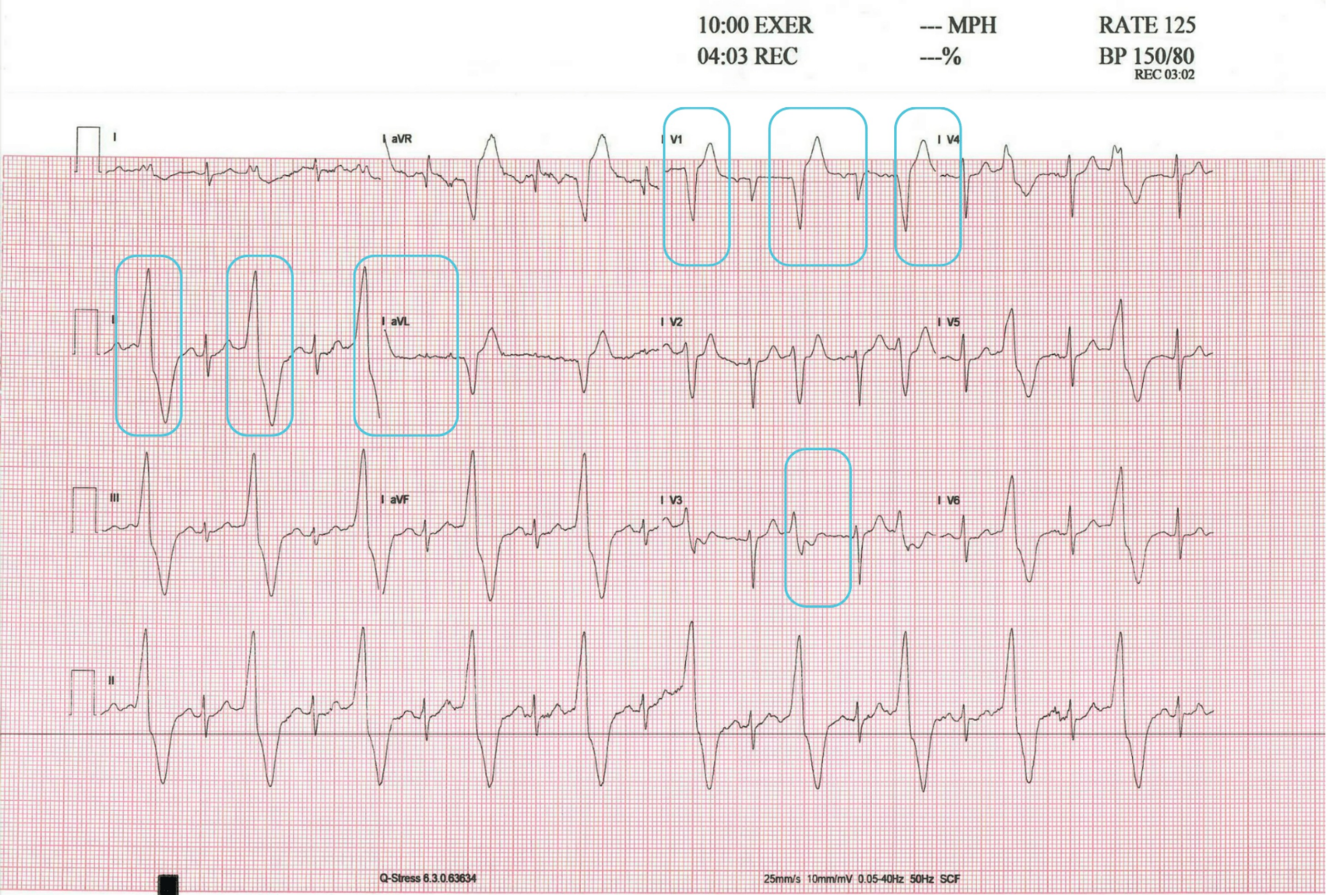
ECG results on the day of diagnosis ECG rhythm strip obtained at 4:03 minutes into the recovery phase of a Bruce protocol treadmill stress test. The tracing demonstrates sinus tachycardia at approximately 125 bpm with ventricular bigeminy. The ectopic QRS complexes display an LBBB-like morphology with an inferior axis and precordial transition in lead V3, findings consistent with an RVOT origin. LBBB, left bundle branch block; RVOT, right ventricular outflow tract

The ECG rhythm strip (leads II, III, aVF, V1, V4, and V5) revealed PVCs originating from the right ventricle, as indicated by a left bundle branch block (LBBB) morphology with an inferior axis, evidenced by positive QRS complexes in leads II, III, and aVF. The PVCs occurred in a bigeminy pattern, as illustrated in Figure 3. Although the patient’s resting ECG was normal, a treadmill stress test was pursued due to his high-risk profile, which included poorly controlled diabetes and an elevated resting heart rate observed during wearable-based remote monitoring.

24-Hour Holter Monitor Data

A 24-hour Holter monitor was indicated as a crucial diagnostic tool to assess the “PVC burden,” referring to the number of premature ventricular beats recorded over 24 hours, as well as their complexity, prognostic significance, and potential to induce left ventricular dysfunction. The patient was continuously monitored for 24 hours, during which the basal rhythm was sinus. The average heart rate was 84 bpm, with a minimum of 61 bpm and a maximum of 150 bpm recorded at 15:27. A total of 2,078 ventricular events were captured, representing 1.75% of all beats. These included 5 isolated events, 254 bigeminy cycles, and 44 trigeminy cycles; no couplets or ventricular runs were noted. Clinically, this burden is considered low, remaining well below the 10% threshold generally associated with PVC-induced cardiomyopathy. However, in a patient with diabetes and multiple cardiovascular risk factors, even a relatively low burden may still justify close monitoring and lifestyle intervention.

Additionally, five isolated supraventricular events were recorded, with no pauses or atrioventricular block observed. PVCs were more frequent during daytime hours and were noted at rest as well as following physical activity. Although the ventricular event percentage remained low and no high-risk patterns such as couplets or ventricular tachycardia were detected, the presence of more than 500 PVCs within 24 hours supports the need for ongoing observation.

Follow-up monitoring using the ŌURA ring demonstrated favorable trends in autonomic function. The patient’s resting heart rate decreased from 83 bpm to 74 bpm, while HRV, measured via RMSSD, improved from 15.2 ms to 32.8 ms over a two-month period. These improvements indicate enhanced autonomic balance and correlate with the patient’s report of fewer symptomatic episodes. Although not a replacement for repeat Holter monitoring, the wearable-derived metrics supported the clinical decision to pursue continued nonpharmacologic management and defer therapeutic escalation.

Differential Diagnosis

The differential diagnosis of frequent PVCs in diabetic patients requires a comprehensive evaluation that includes a detailed clinical history, physical examination, ECG, Holter monitoring, echocardiography, and, in selected cases, cardiac MRI.

The initial assessment focused on excluding inherited arrhythmogenic syndromes and identifying any family history of sudden cardiac death. The patient did not report episodes of syncope or symptoms suggestive of ventricular tachycardia, arrhythmogenic right ventricular dysplasia, or other inherited cardiomyopathies. Additionally, there was no known family history of arrhythmias or sudden cardiac death. In the absence of concerning symptoms, abnormal cardiac imaging, or suggestive family history, genetic testing was not pursued, consistent with current guidelines recommending such testing only in higher-risk scenarios.

To rule out underlying structural heart disease, the baseline ECG was examined for signs of depolarization or repolarization abnormalities, including pathological Q waves, QRS fragmentation, and T-wave inversions. This step is particularly important in diabetic patients due to their increased risk of silent myocardial ischemia and infarction, which can alter ventricular conduction and increase the incidence of PVCs. In this case, the baseline ECG was normal and showed no evidence of ischemic heart disease.

An echocardiogram was then performed to evaluate for systolic dysfunction potentially related to a high PVC burden or diabetic cardiomyopathy. Diabetic cardiomyopathy can result in structural changes such as ventricular hypertrophy and fibrosis, predisposing patients to arrhythmias. However, the echocardiogram demonstrated normal biventricular systolic function and no signs of diabetic cardiomyopathy. Other potential contributors, including medications, electrolyte imbalances, and autonomic nervous system dysfunction, were also considered. However, no supporting evidence for these conditions was found.

With these possibilities excluded, the focus shifted to evaluating the morphology of the PVCs, which can provide insights into their origin. The patient’s PVCs were diagnosed as idiopathic right ventricular outflow tract (RVOT) in origin. These typically exhibit an LBBB morphology, characterized by a negative QRS complex in lead V1 and a late precordial transition, along with an inferior axis and negative deflections in leads aVL and aVR - features consistent with a vector originating from the base of the heart. In this patient, the PVCs were reduced during the exercise phase of a treadmill stress test but became more pronounced during the recovery period, a pattern commonly seen in RVOT PVCs.

PVCs from the RVOT are often triggered by delayed afterdepolarizations, which result from elevated intracellular calcium levels. In this case, PVCs were more frequent during the day and occurred both at rest and after physical exertion. Caffeine was identified as a potential exacerbating factor. The patient reported consuming more than five cups of coffee per day. Caffeine is known to induce delayed afterdepolarizations by promoting calcium release from the sarcoplasmic reticulum, likely contributing to the increased frequency of PVCs in this case.

Diagnostic assessment

Table 1 summarizes the patient’s clinical parameters at baseline and at the four-month follow-up. While there was overall improvement in the patient’s metabolic profile, fasting glucose showed a modest increase from 122 mg/dL to 136.6 mg/dL. This isolated elevation may be attributed to transient factors such as psychological stress, recent dietary intake, or circadian variation, and does not necessarily reflect a deterioration in glycemic control, particularly given the stable HbA1c values (5.9% at baseline and 5.8% at follow-up). Ongoing monitoring is recommended to determine whether this rise represents a persistent trend.

**Table 1:** Baseline and follow-up clinical characteristics and blood test results ALT, alanine aminotransferase; AST, aspartate aminotransferase; CKD, chronic kidney disease; eGFR, estimated glomerular filtration rate; HbA1c, hemoglobin A1c; HDL, high-density lipoprotein; LDL, low-density lipoprotein

Parameter	Baseline	Four-month follow-up	Reference range
Weight (kg)	103	99.55	-
BMI (kg/m²)	29.7	28.78	18.5-24.9 (normal range)
HbA1c (%)	5.9	5.8	<5.7% (normal), 5.7-6.4% (prediabetes), ≥6.5% (diabetes)
Fasting glucose (mg/dL)	122	136.6	70-99 (normal), 100-125 (impaired), ≥126 (diabetes)
Cholesterol (mg/dL)	134.9	151.2	<199
LDL (mg/dL)	67.7	79.9	<100
HDL (mg/dL)	46	56.4	≥40 (men), ≥50 (women), >60 (protective)
Triglycerides (mg/dL)	87.6	106.6	<149
ALT (U/L)	16.8	22.5	<41
AST (U/L)	12.7	15	<40
eGFR (mL/min/1.73 m²)	145.8	158.62	≥90 (normal), 60-89 (mild decrease), <60 (possible CKD)

Therapeutic intervention

The primary management strategy for this patient focused on nonpharmacological interventions, given his asymptomatic presentation and absence of structural heart disease. The observed PVCs were deemed idiopathic and largely adrenergically mediated, often triggered by physiological stressors such as emotional stress, physical activity, and stimulant use, all of which heighten adrenergic tone and may precipitate arrhythmias. The patient was advised to stop smoking, adopt stress-reduction techniques, and limit caffeine intake to help reduce PVC frequency and improve overall cardiac health. Notably, after decreasing his caffeine consumption, a marked reduction in PVCs was observed, as demonstrated by a normal follow-up ECG and improved HRV trends. The patient was educated on the benign nature of idiopathic PVCs in the absence of structural heart disease and was reassured about the favorable prognosis. The importance of lifestyle modifications was emphasized, with recommendations tailored to his specific risk factors, reinforcing that even modest changes can significantly impact arrhythmia burden and long-term cardiovascular well-being.

Follow-up and outcomes

**Figure 4:**
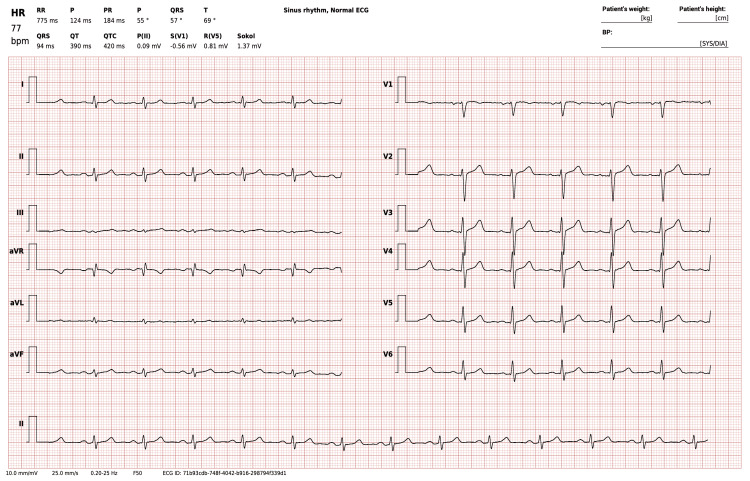
Follow-up ECG results three days after initial diagnosis

Less than 72 hours after implementing the recommended lifestyle changes, HRV data began to show improvement, as illustrated in Figure 4. A follow-up 12-lead ECG conducted three days after diagnosis revealed a regular sinus rhythm with no PVCs or other abnormalities, confirming short-term suppression of arrhythmias following the interventions.

As part of our hybrid care model, diabetic patients are evaluated every three months for standard testing. Cardiology follow-up is tailored based on risk stratification: every three months for symptomatic patients or those with a high PVC burden (>500/day), every six months for moderate-risk individuals (e.g., abnormal ECG or declining HRV), and annually for low-risk, stable patients. These visits help guide decisions on the need for repeat Holter monitoring or echocardiography. Should future monitoring reveal significant changes in PVC frequency or symptom development, pharmacological treatment, such as beta-blockers or nondihydropyridine calcium channel blockers, may be considered.

The integration of the ŌURA ring into this care model ensures a continuous connection between the patient and the healthcare team, regardless of location. The patient’s ŌURA data has shown improvements in HRV and a reduction in arrhythmia-like episodes, supporting the impact of lifestyle modifications. However, these changes remain observational and do not replace formal PVC quantification. Repeat Holter monitoring will be pursued if clinically indicated to confirm long-term progress.

Through the GluCare app, the patient can easily share health data and receive guidance between visits. This ongoing access to real-time physiological data enables the care team to make dynamic, informed adjustments to the treatment plan, ensuring it remains aligned with the patient’s current health status. Regular follow-ups every three to 12 months are recommended to monitor progress and assess the need for repeat diagnostic testing.

## Discussion

The integration of digital wearables into hybrid care models marks a significant advancement in personalized healthcare. These models, which combine traditional face-to-face consultations with digital monitoring and behavioral interventions, offer a comprehensive approach that leverages the strengths of both clinical and remote care settings [6,7]. This approach is particularly relevant for high-risk patients, such as those with diabetes and a history of smoking. In such cases, wearable devices like the ŌURA ring can provide continuous physiological data that healthcare professionals can monitor remotely, thereby extending patient surveillance beyond the clinic.

The use of wearable devices for continuous monitoring has been extensively studied. Tison et al. demonstrated the potential of commercial smartwatches to detect atrial fibrillation through passive monitoring [9], while Steinhubl et al., in the mSToPS trial, showed that home-based wearable ECG patches improved the detection of previously undiagnosed arrhythmias [10]. Furthermore, Cao et al. validated the accuracy of heart rate and HRV data from consumer wearables against ECG readings [11]. These findings support the observations in this case, where shifts in HRV and resting heart rate captured by the ŌURA ring led to early diagnostic evaluation. However, wearables are not without limitations. Data quality may be affected by sensor variability, user compliance, motion artifacts, and physiological states. As Piwek et al. noted, challenges such as inconsistent device use and false positives may contribute to unnecessary diagnostic procedures [12].

To effectively incorporate digital biomarkers into clinical practice, GluCare has introduced several internal protocol adjustments. Clinical staff are now trained to interpret heart rate and HRV trends, with specific thresholds embedded in clinical workflows - for instance, an RMSSD drop of more than 30% or a resting heart rate exceeding 110 bpm prompts further review. Nursing teams receive automated weekly reports, and any flagged cases are escalated for ECG orders, patient outreach, or in-person visits. These protocols ensure that insights from wearable data are actionable and seamlessly integrated into routine care.

This hybrid care model also has the potential to extend beyond cardiology and diabetes. Conditions such as hypertension, menopause, and polycystic ovary syndrome could benefit from the continuous remote monitoring of stress levels, temperature fluctuations, and HRV trends, coupled with personalized behavioral coaching. As wearable technology and artificial intelligence algorithms evolve, improvements in specificity and reliability are expected, paving the way for scalable, proactive management of various chronic diseases in real-world settings.

Despite its promise, the broader implementation of wearable-enabled hybrid care models faces several challenges. These include maintaining patient adherence, addressing variability in device accuracy, ensuring user-friendly technology, training clinicians to interpret digital biomarkers effectively, and mitigating alert fatigue from continuous data streams. This case illustrates the clinical utility of wearable data, specifically, the early identification of decreased HRV and elevated daytime heart rate as a trigger for timely diagnostic escalation. However, the generalizability of these findings remains limited. Further research is needed to compare the effectiveness of different wearable platforms, assess long-term outcomes, and develop strategies to improve adherence. Moreover, while interest in the economic impact of such models is growing, robust evidence on their cost-effectiveness is still lacking and should be a priority in future investigations to support widespread adoption and policy integration.

## Conclusions

The synergy between digital wearables and hybrid care models presents a promising pathway for transforming healthcare delivery. By enabling early detection, supporting continuous patient monitoring, and promoting a more patient-centered approach, these innovations drive a shift toward proactive and preventive care. The encouraging findings from this case study highlight the potential value of integrating wearable technology into routine clinical practice, particularly for individuals with chronic conditions such as diabetes and associated cardiovascular risk. Future research should prioritize large-scale studies to further validate the clinical effectiveness and cost-efficiency of wearable technologies across diverse patient populations and healthcare settings.
